# Comparative Effects of Allulose, Fructose, and Glucose on the Small Intestine

**DOI:** 10.3390/nu14153230

**Published:** 2022-08-07

**Authors:** Takuji Suzuki, Yuki Sato, Sumire Kadoya, Takumi Takahashi, Moeko Otomo, Hanna Kobayashi, Kai Aoki, Mai Kantake, Maika Sugiyama, Ronaldo P. Ferraris

**Affiliations:** 1 Faculty of Agriculture, Yamagata University, Yamagata 997-8555, Japan; 2 Faculty of Education, Art and Science, Yamagata University, Yamagata 990-8560, Japan; 3 Department of Food Science and Nutrition, Faculty of Human Life and Science, Doshisha Women’s College of Liberal Arts, Kyoto 602-0893, Japan; 4 Department of Pharmacology, Physiology & Neuroscience, Rutgers New Jersey Medical School, Newark, NJ 07103, USA

**Keywords:** allulose, fructose, glucose, small intestine, small intestinal function, nutrient digestion and absorption, intestinal barrier

## Abstract

Despite numerous studies on the health benefits of the rare sugar allulose, its effects on intestinal mucosal morphology and function are unclear. We therefore first determined its acute effects on the small intestinal transcriptome using DNA microarray analysis following intestinal allulose, fructose and glucose perfusion in rats. Expression levels of about 8-fold more genes were altered by allulose compared to fructose and glucose perfusion, suggesting a much greater impact on the intestinal transcriptome. Subsequent pathway analysis indicated that nutrient transport, metabolism, and digestive system development were markedly upregulated, suggesting allulose may acutely stimulate these functions. We then evaluated whether allulose can restore rat small intestinal structure and function when ingested orally following total parenteral nutrition (TPN). We also monitored allulose effects on blood levels of glucagon-like peptides (GLP) 1 and 2 in TPN rats and normal mice. Expression levels of fatty acid binding and gut barrier proteins were reduced by TPN but rescued by allulose ingestion, and paralleled GLP-2 secretion potentially acting as the mechanism mediating the rescue effect. Thus, allulose can potentially enhance disrupted gut mucosal barriers as it can more extensively modulate the intestinal transcriptome relative to glucose and fructose considered risk factors of metabolic disease.

## 1. Introduction

D-allulose (allulose), also known as D-psicose, is an epimer at the 3rd carbon of fructose that, relative to glucose and fructose, is found in extremely low concentrations less than about 0.15% in many foods, and thus is considered a ‘rare’ sugar [1]. Despite its low natural abundance, allulose has attracted much attention because of its strong nutraceutical properties. Allulose provided as a dietary supplement exhibits anti-diabetic properties in rats by reducing plasma glucose, increasing insulin secretion, and protecting the morphology and function of pancreatic islets [2,3,4]. The mechanism underlying allulose’s antidiabetic properties is due, in part, to enhanced secretion of glucagon such as peptide-1 (GLP-1) from intestinal epithelial endocrine L cells [5]. GLP-1 is an incretin known to inhibit glucagon and stimulate insulin release [6]. More recent work also suggests that allulose acts on vagal afferents to inhibit feeding and dampen hyperglycemia [7], and on hepatocytes to suppress de novo lipogenesis thereby decreasing levels of triacylglycerol and free fatty acids in blood [8,9,10]. Allulose is also nontoxic, even if consumed by rats for 18 months [11]. In humans, allulose absorbed into the body is excreted into the urine without being metabolized [12].

Because its chemical structure is similar to fructose, allulose is presumed to be absorbed via the intestinal fructose transporter GLUT5. Allulose was absorbed via GLUT5 in Caco-2 cells in vitro and in rat intestine in vivo, although in vitro competition studies suggest allulose can also enter mouse intestinal cells via the GLUT5 [13,14]. Although demonstrated to be absorbed by the small intestine and to stimulate intestinal enteroendocrine cells to secrete GLP-1, the effects of allulose on other functions of the small intestine are unknown. Moreover, there may be other mechanisms underlying its known anti-hyperglycemic and anti-hyperlipidemic effects as well as proposed anti-inflammatory and anti-hyperphagic properties [15,16,17].

To characterize the effects of allulose on the small intestinal epithelial cells, this study first analyzed its effects, compared to those of glucose or fructose, using DNA microarray analysis following intestinal luminal perfusion, an experimental method that directly exposed the cells to glucose, fructose or allulose only, without the confounding effects of interactions with other nutrients and with the gut microbiota. The microarray analysis has the advantage of a high throughput, untargeted and unbiased approach to assessing the intestinal effects of allulose when compared to typical sugars glucose and fructose. On the other hand, total parenteral nutrition (TPN) typically reduces intestinal mucosal growth and absorptive area [18], but can be restored by oral gavage of glucose or fructose [19]. We thus next investigated whether allulose actually has the same mucosal growth-stimulating potential as that of glucose and fructose. In addition, in order to determine the underlying factors of action of various monosaccharides in stimulating small intestinal function, we analyzed using normal mice the effects of various monosaccharides gavage on the secretion of the GLP-2 and the insulin-like growth factor, IGF, which are involved in homeostasis of villi function in the small intestine. This study is the first to demonstrate that allulose, which is not metabolized by the body, has the ability to maintain not only the expression of genes involved in a wide variety of functions but also structure of crypts and villi, which is similar to or exceeds that of widely consumed sugars, glucose and fructose, considered risk factors of metabolic disease.

## 2. Materials and Methods

Materials. Glucose and fructose were purchased from Kanto Chemical Co., Inc. (Tokyo, Japan). Purified allulose were supplied from Matsutani Chemical Industry Co., Ltd. (Osaka, Japan). 

Animals. For studies on sugar perfusion and total parenteral nutrition in rats. The animal research ethics committee of Yamagata University approved these studies (approval number 26143, 27051, 28042 and 31167). Nine weeks-old Wistar strain male rats were purchased from Japan SLC, Inc. (Hamamatsu, Shizuoka, Japan). All animals were acclimatized before experiments under standard vivarium conditions for 7 days. Rats were housed on a 12-h light-dark cycle with ad libitum access to a standard diet (CRF-1; Oriental Yeast, Tokyo, Japan) and water before any experiment. 

For the study on gastrointestinal hormone measurement in normal mice. The animal research ethics committee of Doshisha Women’s College of Liberal Arts approved this study (approval number S21-008). Nine weeks-old ICR strain male mice were purchased from Japan SLC, Inc. All animals were acclimatized before experiments under standard vivarium conditions for 7 days. Mice were housed on a 12-h light-dark cycle with ad libitum access to a standard diet (MF; Oriental Yeast) and water before experiments. 

Intestinal sugar perfusion. This method was modified from that of Jiang and Ferraris [20]. Rats were anesthetized by intraperitoneal injection of 1000 mg/kg body weight urethane (250 mg/mL urethane in distilled water). The abdominal cavity was opened, and the small intestine with intact blood vessels and nerve connections was exposed. About 1 cm distal to the pylorus to the ligament of Treitz, a small incision was made, and 18G stainless tube, fitted into the one side of the silicon tube (Laboran^®^ silicone tube, 1.0 mm inner diameter, AS ONE Corporation, Osaka, Japan), inserted in the lumen and then secured with surgical thread. The other side of silicone tube was connected to 18G injection needle cut off the needle tip and fitted to plastic syringe. To discharge perfusion solution from intestinal tract, a small incision was made at the end of the ileum. After the contents were flushed by Krebs-Ringer bicarbonate buffer (KRB, pH 7.4 containing 0.49 mM magnesium chloride, 120 mM sodium chloride, 4.56 mM potassium chloride, 0.70 mM sodium phosphate dibasic, 1.30 mM sodium phosphate monobasic, 15 mM sodium bicarbonate), the small intestine was continuously perfused for 5 h with 60 mM sugar solution (1.0% (*w*/*v*) glucose, fructose or allulose in KRB), at a rate of 60 mL/h at 37 °C using syringe pump (YSP-101, YMC Co., Ltd., Kyoto, Japan). Rats were kept under continuous anesthesia by adding urethane solution into the abdominal cavity every 30 min. Temperatures of the body using electric panel heater (Super 1, Midori Shoukai, Tokyo, Japan) and perfusion solution using water bath were maintained at 37 °C, respectively. 

Preparation of jejunal samples for DNA microarray analysis and gene expression analysis. After intestinal sugar perfusion for 5 h. The entire small intestine was removed, and the jejunoileum was isolated and flushed with ice-cold 0.9% (*w*/*v*) NaCl in 0.1% (*v*/*v*) diethyl pyrocarbonate (DEPC) treated distilled water. The proximal one-third of the jejunoileum was considered as the jejunum, and 3 cm at approximately the central position of the jejunum was used for DNA microarray analysis and gene expression analysis. Separated jejunal segment was cut open on the cold glass dish. Then, intestinal mucosa was scraped using slide glass and put in RNAlater™ (Thermo Fisher Scientific, Waltham, MA, USA) and stored at −80 °C. 

Total parenteral nutrition (TPN) treatment and jejunal sample preparation. The methods for TPN surgery have been previously described [19]. The sham animals (used as controls for the TPN-treated animals) were given a standard diet and water ad libitum until the completion of the study following surgery (*n* = 4). TPN-treated animals (TPN group) were administered commercially TPN liquid (NEOPAREN. No. 2 injection, Otsuka Pharmaceutical Factory, Inc., Tokushima, Japan) equating to a total calorie count of 250 kcal/kg BW/day via a catheter inserted into the jugular vein for 7 days. The administered total calorie amount was set at an amount that did not significantly reduce the animals’ body weight by our previous investigation. The TPN-treated animals were subsequently euthanized by bleeding from inferior vena cava under the isoflurane (Wako Pure Chemical Ind., Ltd., Osaka, Japan) anesthesia and dissected. In another group, rats were treated with TPN for 7 days and were subdivided into three groups, where one group was administered 3 g/kgBW/day glucose (Glucose group; *n* = 4), the second group was given 3 g/kgBW/day fructose (Fructose group; *n* = 4) and the third group was given 3 g/kgBW/day allulose (Allulose group; *n* = 4). These three groups were given an aqueous solution containing 15% monosaccharides (*w*/*w*) orally once a day for 2 days, and deficient nutrients continued to be supplemented by TPN infusions. The TPN group was dissected 7 days post-TPN liquid cannulation. Nine days post start of the experiments, the Sham, Glucose, Fructose and Allulose groups were dissected. All animals were euthanized under isoflurane anesthesia. The entire small intestine was removed, and the duodenum extending from the pylorus to the ligament of Treitz was isolated, and the inside of the jejunoileal tract was flushed with ice-cold 0.9% NaCl solution. The proximal one-third of the jejunoileum was assigned the jejunum, and 1 cm at the approximate center position of the jejunum was used for histochemical staining. The remaining jejunal samples were used for gene and protein expression analysis and the measurement of disaccharidase activity. All samples were frozen in liquid nitrogen and stored at −80 °C for later analysis. 

Total RNA extraction. Total RNA was extracted from the jejunal mucosa using TRIzol™ Reagent (Thermo Fisher Scientific). Extracted total RNA was used for DNA microarray analysis and quantitative RT-PCR (qRT-PCR). 

DNA microarray analysis and additional analysis. DNA microarray analysis was consigned to Hokkaido System Science Co., Ltd. (Sapporo, Hokkaido, Japan) SurePrint G3 Rat 8 × 60 K ver.2.0 (Agilent Technologies, Santa Clara, CA, USA) was used for one color microarray analysis. Each total RNA sample passed RNA quality check (Glucose; *n* = 2, Fructose; *n* = 3 and Allulose; *n* = 3) prior to conversion to cDNA. The entire methods of DNA microarray analysis such as cRNA labeling, hybridization, washing, scanning, digitization, and normalization followed standard protocols of the company. Clustering, Venn diagram and fold change (+2-fold or more in gene expression) were also analyzed. The transcriptome was analyzed using Ingenuity^®^ Pathway Analysis (IPA, Hilden, Germany, QIAGEN). The methods of analysis were according to manufacturer’s instruction. 

qRT-PCR. An amount of 500 ng total RNA was then converted to cDNA by reverse transcription using a Takara prime RT reagent Kit (TakaraBio, Kusatsu, Shiga, Japan) for qRT-PCR. A qRT-PCR was performed to quantify the mRNA levels of target genes using an ABI 7500 real-time PCR system (Life Technologies, Carlsbad, CA, USA). PCR was performed with the SYBR Premix Ex Taq II (Tli RNase H Plus, TakaraBio); primers sequences are listed in supplementary table (Table S1). The cycle threshold (CT) values obtained by qRT-PCR analysis and mRNA levels were calculated using CT values based on the ^ΔΔ^Ct method.

Morphological analysis. Jejunal tissue was immediately immersed in a 10% formaldehyde neutral buffer solution (Nacalai Tesque, Kyoto, Japan) and stored at 4 °C. Fixed samples were embedded in paraffin (GemCut Emerald Paraffin, Polysciences Inc., Warrington, PA, USA), then sectioned into 4-μm thick slices, and stained with hematoxylin and eosin (HE). Hematoxylin and Eosin staining was conducted as per the standard method using Mayer’s hematoxylin solution (Wako Pure Chemical Ind., Ltd.) and 1% eosin Y solution (Nacalai tesque). Alcian blue and periodic acid-SCIFF (PAS) double staining was performed, with alcian blue staining conducted first followed by PAS staining according to standard method. All slides were subjected to a dehydration process using ethanol and xylene and then mounted by mounting media (Multi mount 480, Matsunami Glass Ind., Ltd., Osaka, Japan). These tissue sections were then imaged (BA210E, Shimazu Corporation, Kyoto, Japan, 100×). Villous height (VH), crypt depth (CD), and muscle layer thickness were measured using image analysis software (Motic Image Plus 2.3s, Shimadzu Rika Corporation, Tokyo, Japan). A turnover ratio (TR) was calculated by dividing VH by CD (VH/CD).

Immunoblot analysis. Total proteins were extracted from jejunal samples using radioimmunoprecipitation assay (RIPA) buffer (1% NP-40, 0.1% SDS, 20 mM Tris-HCl (pH 8.0), 5 mM EDTA, 150 mM NaCl with proteinase inhibitor tablets (Complete™ mini, Roche, Basel, Switzerland) and phosphorylase inhibitor tablets (PhosSTOP™, Roche, Basel, Switzerland). Total protein concentrations for each sample were calculated by BCA protein assay Kit (Thermo Fisher Scientific). Each protein sample was prepared to a total protein concentration of 5.0 μg/μL for each animal per treatment. Each protein sample of the Sham group was pooled to ensure the same mixing ratio (*n* = 4). On the other hand, we used each protein sample of the other treatment groups extracted from each individual without pooling (*n* = 3). 15 μg/lane of total protein was loaded onto a 10% SDS-polyacrylamide gel electrophoresis. For the immunoblot analysis, the primary antibodies used anti-sucrase-isomaltase (sc-393470, Santa Cruz Biotechnology, TX, USA), anti-I-FABP (sc-376070, Santa Cruz Biotechnology), anti-ZO-1 (21773-1-AP, Proteintech Group Inc. Japan, Tokyo, Japan), anti-occludin (27260-1-AP, Proteintech Group Inc., Rosemont, IL, USA) and anti-TFIIB (sc-225, Santa Cruz Biotechnology). Anti-rabbit IgG, HRP-linked antibody (W4018, Promega corporation, Fitchburg, WI, USA) or Anti-mouse IgG, HRP-linked antibody (W4028, Promega corporation) was used as secondary antibodies. Proteins were detected by chemiluminescence reagent (SignalFire™ ECL Reagent, Cell Signaling Technology, Inc., Danvers, MA, USA). Protein blots were imaged using a Lumino Graph I (WSE-6100H, ATTO, Tokyo, Japan). The detected bands were quantified using Image J software which is provided by the National Institutes of Health.

Immunohistochemistry. Fixed samples were embedded in paraffin (GemCut Emerald Paraffin, Polysciences Inc.), then sectioned into 4-μm thick slices. Slide glasses with sliced jejunal sections were boiled at 85 °C for 20 min in HistoVT One (Nacalai tesque) to activate the antigen. Then, the samples were washed well with PBS and blocked by Blocking One (Nacalai tesque) for 20 min at room temperature. Next, 100-fold dilutions of each primary antibody; anti-ZO-1 (21773-1-AP, Proteintech Group Inc., Rosemont, IL, USA) and anti-occludin (21773-1-AP, Proteintech group Inc. Rosemont, IL, USA) were made in 20-fold dilutions of Blocking One. These slides were incubated with each primary antibody at 4 °C overnight and then washed in T-TBS (PBS with 0.1% Tween 20). After washing, secondary antibody (Goat anti-Rabbit IgG H + L Highly Cross-Absorbed Secondary antibody with Alexa Fluor Plus 488, Thermo Fisher Scientific) diluted 20-fold by Blocking One was diluted to 1000-fold and incubated to react for 1.5 h at room temperature. After washing, nuclei were stained with 1 μg/mL of DAPI solution for 15 min. After sealing with antifade regent (ProLong™ Glass Antifade Mountant, Thermo Fisher Scientific), the slides were dried overnight under light-shielded conditions. Stained tissue sections were observed and photographed under a fluorescence microscope (IX73 with DP74 camera system, Olympus, Tokyo, Japan).

Disaccharidase activity assay. Each jejunum intestinal mucosa sample was homogenized using 10 mM PBS (containing 10 mM potassium dihydrogen phosphate and 10 mM dipotassium hydrogen phosphate, pH 7.0) using a homogenizer (HOM, AS ONE Corporation). Homogenates were used for the disaccharidase activity assays according to the method developed by Dahlqvist [21]. The modified method has been previously described [19,22].

Measurements of GLP-2, IGF-2, and GLP-1 in plasma. In the case of TPN study, the blood samples were collected from the inferior vena cava of each treated rat (*n* = 4) under isoflurane anesthesia at dissection. Likewise, the blood samples were collected from the portal vein of male ICR mice fasted overnight (18:00 to next 10:00) under isoflurane anesthesia at 30 min after oral administration of glucose, fructose and allulose (15 mmol/ kgBW, 10 mL/kgBW) or saline (10 mL/kgBW), respectively (*n* = 6). The sampling syringe contained heparin (final concentration; 50 IU/mL), aprotinin (final concentration; 500 kIU/mL), and DPP-IV inhibitor (DPP4, Merck, Darmstadt, Germany, final concentration; 50 µM). Plasma was collected after centrifugation (3000× *g*, 10 minutes at 4 °C) and stored at −80 °C until assay. Total GLP-2 were measured using Rat GLP-2 EIA Kit (YK140, Yanaihara Institute, Inc., Shizuoka, Japan) or Mouse GLP-2 EIA Kit (YK142, Yanaihara Institute, Inc.). IGF-2 were measured using Mouse/Rat/Porcine/Canine IGF-II/IGF2 Quantikine™ ELISA Kit (MG200, R&D systems, Acton, MN, USA). Total GLP-1 were measured using GLP-1 ELISA kit (EZGLP1T-36K, Merck).

Statistical analysis and graph creation. Data are shown as the mean ± SEM. A post hoc analysis used Dunnett’s multiple comparisons test following One-way analysis of variance (ANOVA). A significant difference was considered any value of *p* < 0.05. Statistical analysis and graph creation were performed using GraphPad Prism 8.4.3 software for Windows (GraphPad Software Inc., San Diego, CA, USA).

## 3. Results

### 3.1. DNA Microarray Analysis (Clustering Analysis, Venn Diagram Analysis and Transcriptome Analysis)

**Figure 1:**
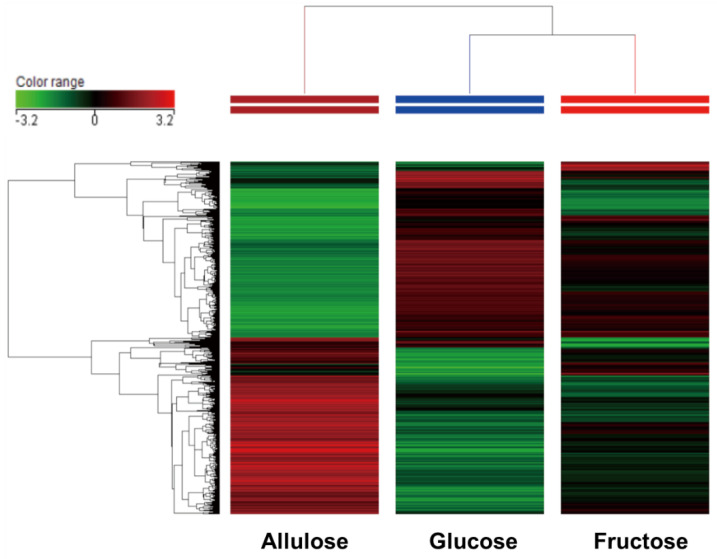
Clustering analysis.

**Figure 2:**
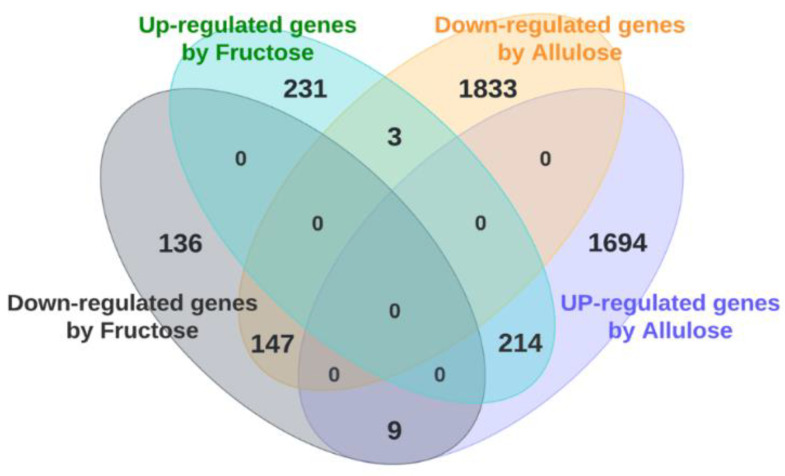
Venn diagram analysis.

A total of 20,551 genes were subjected to clustering heat map analysis which showed three clearly different expression patterns. Gene expression was more similar between fructose- and glucose-perfused intestines compared to allulose-perfused ones (Figure 1). About 45% of the genes were markedly upregulated by allulose compared to similar sections in those glucose- and fructose-perfused. Another 45% was downregulated by allulose. About 10% of genes increased dramatically in glucose- or fructose-perfused intestines, compared to allulose-perfused intestines. Venn diagram analysis using fold-change data of the microarray analysis showed that 234 genes were up-regulated and 145 genes were down-regulated more than 2.0-fold in fructose-perfused animals compared to glucose-perfused control animals. On the other hand, allulose-perfused animals had increased expression levels of 1703 genes and decreased expression levels of 1836 genes. A total of 214 genes were simultaneously upregulated by fructose and allulose, relative to glucose-perfusion, while 147 genes were simultaneously downregulated by fructose and allulose (Figure 2). Thus, relative to fructose and glucose, allulose impacts the expression of a greater number of genes, potentially signifying a greater allulose effect on intestinal physiology.

To characterize the effects of allulose on the small intestinal epithelial cells, we performed transcriptome analysis using Ingenuity^®^ Pathway Analysis (IPA). Consistent with the clustering and Venn analysis, a greater number of functional categories seemed to be associated with allulose-perfusion than with fructose-perfusion, compared to glucose-perfusion. Numerous alterations in molecular/cellular function and physiological system development/function categories were associated with allulose exposure, including infectious disease, inflammatory response, molecular transport, protein synthesis, connective tissue development/function and digestive system development/function etc. There were 17 categories that were predicted to be upregulated by allulose-perfusion, while 12 were predicted to be downregulated (Table 1A,B). Small intestinal morphology and function, carbohydrate metabolism, lipid metabolism, molecular transport and cellular assembly and organization were markedly activated with allulose perfusion compared to glucose, while many categories related to inflammatory response showed a marked inhibition by allulose.

Results clearly indicate three different trends of gene expression, with transcriptome of intestines perfused with glucose being more related to that of fructose-perfused, and intestines perfused with allulose markedly different from both glucose- and fructose-perfused. There are also two large groups of transcripts, with those downregulated with allulose being more closely related to each other compared to those upregulated.

The images are drawn as Venn diagram using DNA microarray data. Numbers in each circle indicate number of genes whose expression levels have changed more than two-fold (up or downregulated) by fructose or allulose perfusion compared to glucose perfusion as the control.

### 3.2. Expression of Representative Genes Related to Digestive System Development and Function

**Figure 3:**
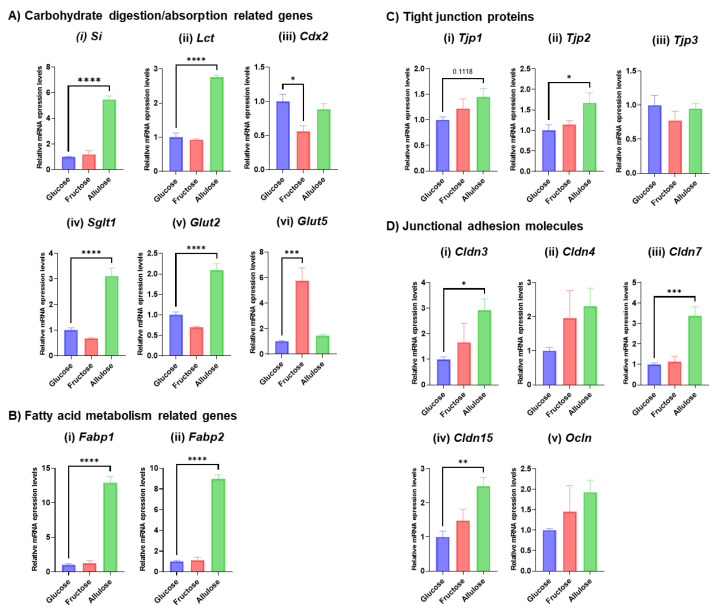
Gene expression levels of representative genes related to nutrient digestion and absorption, and intestinal barrier. (**A**) Results of qRT-PCR for carbohydrate digestion/absorption-related genes. (**i**) *Si*, (**ii**) *Lct*, (**iii**) *Cdx2*, (**iv**) *Sglt1*, (**v**) *Glut2* and (**vi**) *Glut5*. (**B**) Results of qRT-PCR for fatty acid metabolism-related genes. (**i**) *Fabp1* and (**ii**) *Fabp2*. (**C**) Results of qRT-PCR for tight junction proteins. (**i**) *Tjp1*, (**ii**) *Tjp2* and (**iii**) *Tjp3*. (**D**) Results of qRT-PCR for junctional adhesion molecules. (**i**) *Cldn3*, (**ii**) *Cldn4*, (**iii**) *Cldn7*, (**iv**) *Cldn15* and (**v**) *Ocln*. Values indicate mean ± SEM (*n* = 4). Asterisks indicate significant differences tested using the ANOVA-Dunnett’s multiple comparisons test (vs. glucose-perfused group, **** *p* < 0.0001, *** *p* < 0.001, ** *p* < 0.01, * *p* < 0.05).

Because we found carbohydrate metabolism, and more specifically transcripts, involved in sugar absorption to be upregulated by allulose (Table 1A), expression levels of carbohydrate digestion and absorption related genes, i.e., sucrase isomaltase (*Si*), lactase (*Lct)*, sodium-glucose cotransporter (*Sglt1*), glucose transporter 2 (*Glut2*), fructose transporter (*Glut5*) and caudal type homeobox 2 (*Cdx2*), which a common transcriptional factor regulating *Si* and *Lct* genes [23] were determined by qRT-PCR to confirm IPA predictions. Indeed, the expression of many of these genes was upregulated in the allulose-perfused animals compared to the glucose-perfused animals. On the other hand, the expression level of *Glut5* was significantly induced in fructose- but not in allulose-perfused animals, consistent with our earlier findings showing fructose to be the most potent inducer of its own transporter [24,25]. *Cdx2* expression was significantly decreased in fructose compared to glucose-perfused animals but did not differ between glucose- and allulose-perfused animals (Figure 3A). The IPA prediction that fatty acid metabolism is upregulated by allulose (Table 1A) was confirmed as liver type fatty acid binding protein (*Fabp1*) and intestinal type fatty acid binding protein (*Fabp2*) expression increased (Figure 3B). We also confirmed expression levels of representative genes related to cell-to-cell signaling and interaction, and organismal development (i.e., tight junction proteins, claudins and occluding occludin) predicted to be upregulated by IPA analysis (Table 1A). The expression levels of *Tjp1* and *Tjp2* increased in allulose- compared to glucose-perfused animals, but the expression levels of *Tjp3* genes did not change in any groups (Figure 3C). The expression levels of claudins and occludin genes showed a significant increase or increasing trend with allulose-perfusion (Figure 3D). In summary, the expression of genes belonging to pathways predicted to be upregulated by IPA generally also increase.

Sham animals (used as controls for the TPN-treated animals) were given a standard diet and water ad libitum until the completion of the study following sham surgery (*n* = 4). TPN-treated animals were administered TPN liquid via a catheter inserted into the jugular vein for 7 days. Rats were treated with TPN for 7 days and were subdivided into three groups, and each group was administered 3 g/kgBW/day each monosaccharide (glucose, fructose or allulose (each *n* = 4). These three groups were given an aqueous solution containing 15% monosaccharides (*w*/*w*) orally once a day for 2 days, while still receiving adequate nutrition by TPN infusions. The TPN group was dissected 7 days post-TPN liquid cannulation (*n* = 4). Nine days after start of the experiments, the Sham, Glucose, Fructose and Allulose groups were dissected.

### 3.3. Evaluating the Potential of Allulose in Rescuing TPN-Induced Mucosal Atrophy

**Figure 4:**
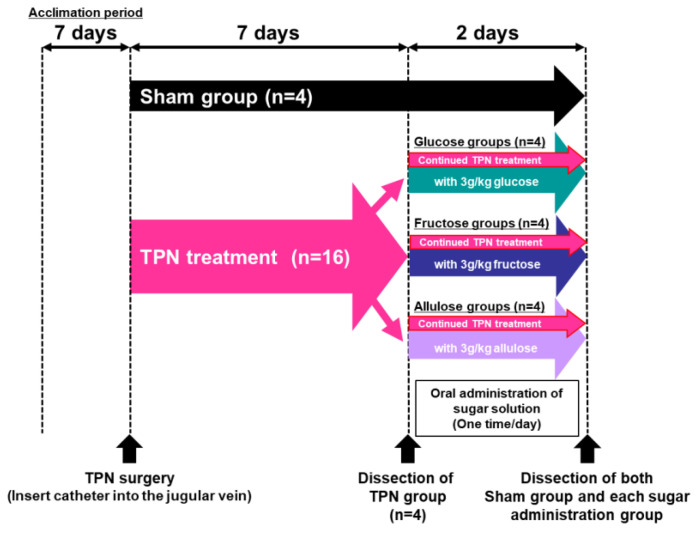
Schematic diagram of TPN study procedure.

**Figure 5:**
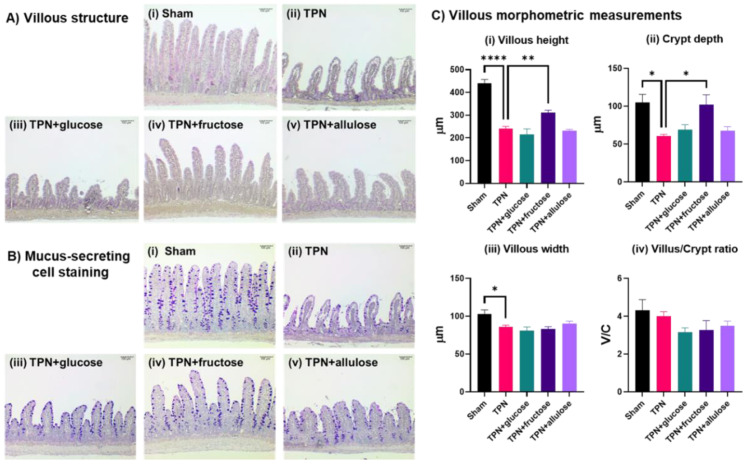
Villous structure and number of mucus-positive cells. (**A**): villous structure of (**i**) sham, (**ii**) TPN, (**iii**) TPN + glucose, (**iv**) TPN + fructose and (**v**) TPN + allulose rats. (**B**): mucus-secreting cell staining (alcian blue-PAS double staining) of jejunum (100×, bar = 100 μm). The images are representative tissue of each group. (**C**): Villous morphometric measurements of (**i**) villous heights, (**ii**) crypt depth, (**iii**) villous width and (**iv**) villus/crypt ratio. Values indicate mean ± SEM (*n* = 4, 7–9 villi/animal). Asterisks indicate significant differences tested using the ANOVA-Dunnett’s multiple comparisons test (vs. TPN group, **** *p* < 0.0001, ** *p* < 0.01, * *p* < 0.05).

Since allulose activated gene pathways related to morphology and function, we investigated its potential to rescue small intestinal structure and function typically reduced after TPN [18,26]. The schematic diagram of TPN study procedure indicated in Figure 4. As expected, villous height, villous width, and crypt depth were significantly decreased in jejunum of the TPN group compared to the Sham group. Decreases in number of goblet cells were also observed in jejunum of the TPN group. On the other hand, in fructose-administered group, villous height and crypt depth were significantly increased compared to the TPN group. Significant alterations on villous morphology were not observed in both the glucose- and allulose-administered groups (Figure 5).

### 3.4. Effect of Monosaccharide Supplementation on Nutrient Metabolism and Transport Genes in TPN

**Figure 6:**
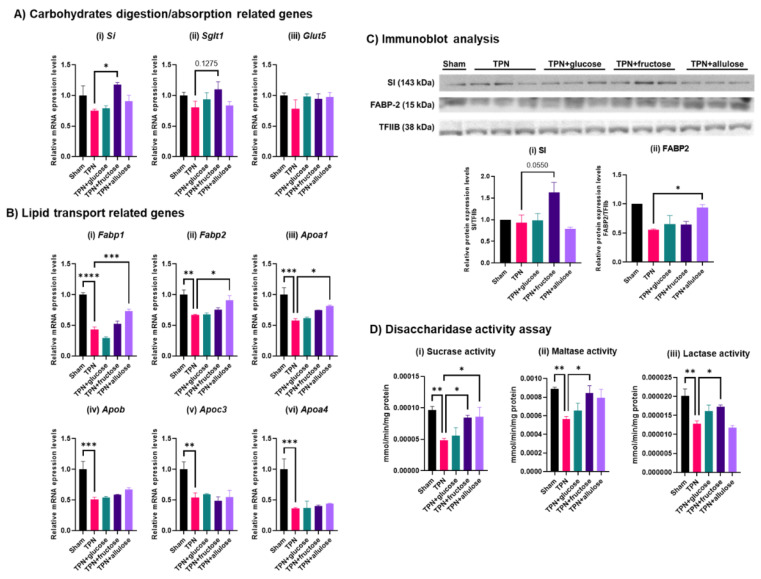
mRNA expression and protein and activities of representative genes involved in digestion and absorption of nutrients in TPN model of rats. (**A**) Results of qRT-PCR for carbohydrates digestion/absorption relate genes, (**i**) *Si*, (**ii**) *Sglt1* and (**iii**) *Glut5.* (**B**) Results of qRT-PCR for lipid transport related genes, (**i**) *Fabp1*, (**ii**) *Fabp2*, (**iii**) *Apoa1*, (**iv**) *Apob*, (**v**) *Apoc3* and (**vi**) *Apoa4*. Values indicate mean ± SEM (*n* = 4). (**C**) Results of immunoblot analysis for sucrase-isomaltase complex (SI) and fatty acid binding protein 2 (FABP2). Transcriptional factor IIB (TFIIB) is internal control. The graphs indicate relative protein expression levels when the value of Sham group is 1.0., (**i**) SI and (**ii**) FABP2. Values indicate mean ± SEM (*n* = 3). (**D**) Results of disaccharidase activity assay. (**i**) sucrase activity, (**ii**) maltase activity and (**iii**) lactase activity. Values indicate mean ± SEM (*n* = 4). Asterisks indicate significant differences tested using the ANOVA-Dunnett’s multiple comparisons test (vs. TPN group, **** *p* < 0.0001, *** *p* < 0.001, ** *p* < 0.01, * *p* < 0.05).

Fructose gavage was observed to modestly recover villus morphology from after TPN treatment, whereas no significant recovery was observed with allulose and glucose gavage. However, since dramatic effects by allulose was observed in the previous experiment on the expression of representative genes involved in nutrient metabolism and transport, here we analyzed the expression and activity of representative genes related to this function in jejunal epithelial cells. *Si* and *Sglt1* expression increased mainly in the fructose administered TPN group (Figure 6A). In contrast to carbohydrate-related genes, there were striking effects of TPN on genes involved in lipid metabolism. mRNA expression of *Fabp1* and *Fabp2*, which play a pivotal role in intracellular lipid transport, as well as of *Apoa1*, a lipoprotein, increased significantly with allulose administration (Figure 6B). Allulose, glucose and fructose did not rescue even modestly expression of other lipoproteins *Apob*, *ApoC3* and *Apoa4*. SI protein expression, like that of mRNA, tended to increase with fructose. Like mRNA, FABP2 protein significantly increased with allulose (Figure 6C), suggesting that its effect may be more pronounced at the protein (translational) level. The activities of three representative disaccharidases, i.e., sucrase, maltase and lactase activities, were modestly upregulated by fructose and sometimes allulose (Figure 6D). In summary, while none of the monosaccharides were able to completely rescue mRNA expression of nutrient absorption and metabolism genes, fructose and allulose supplementation during TPN did rescue their protein expression and function.

### 3.5. Evaluating the Potential of Allulose in Rescuing TPN-Induced Alterations in the Gut Barrier

**Figure 7:**
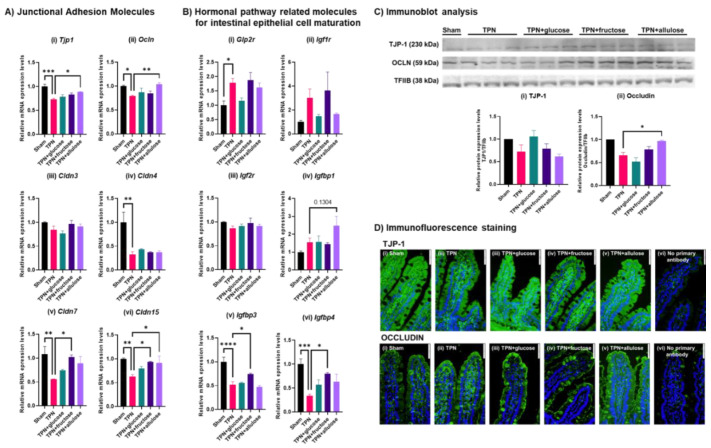
Expression analysis of representative molecules involved in intestinal barrier function and hormonal pathway molecules related to intestinal epithelial cells maturation in TPN model of rats. (**A**) Results of qRT-PCR for junctional adhesion molecules-related genes, (**i**) *Tjp1*, (**ii**) *Ocln*, (**iii**) *Cldn3*, (**iv**) *Cldn4*, (**v**) *Cldn7* and (**vi**) *Cldn15*. (**B**) Results of qRT-PCR for hormonal pathway-related molecules for intestinal epithelial cell maturation related genes, (**i**) *Glp2r*, (**ii**) *Igf1r*, (**iii**) *Igf2r*, (**iv**) *Igfbp1*, (**v**) *Igfbp3* and (**vi**) *Igfbp4*. Values indicate mean ± SEM (*n* = 4). (**C**) Results of immunoblot analysis for tight junction protein 1 (TJP-1) and occludin (OCLN). Transcriptional factor IIB (TFIIB) is internal control. The graphs indicate relative protein expression levels when the value of Sham group is 1.0. Values indicate mean ± SEM (*n* = 3). Asterisks indicate significant differences tested using the ANOVA-Dunnett’s multiple comparisons test (vs. TPN group, **** *p* < 0.0001, *** *p* < 0.001, ** *p* < 0.01, * *p* < 0.05). (**D**) Results of immunofluorescence staining in jejunal sectional samples, (**i**) Sham, (**ii**) TPN, (**iii**) TPN + glucose, (**iv**) TPN + fructose, (**v**) TPN + allulose and (**vi**) no primary antibody for method control. The images were photographed at 400× magnification. The images are representative tissue of each group. The white scale bar at the top right of each image indicates 50 μm. Upper images indicate TJP-1 (green) and nucleic staining by DAPI (blue). Lower images indicate Occludin (green) and nucleic staining by DAPI (blue).

We next analyzed the expression levels of representative genes involved in junctional adhesion and hormonal pathways related to the intestinal barrier and villus morphological maintenance functions. The expressions of almost all junctional adhesion genes were significantly decreased in TPN. Interestingly, allulose seemed to rescue the mRNA expression of many TJ genes, notably *Tjp1*, *Ocln*, *Cldn15* and modestly, *Cldn7* and the protein expression of OCLN. *Cldn7* and *Cldn15* expression also increased with fructose, but glucose had no effect. Glucagon-like peptide-2 (GLP-2) is known as a gastrointestinal hormone related to villus morphological and functional maintenance. Insulin-like growth factors, i.e., IGF-1 and IGF-2 are hormones involved in the villus morphological and functional maintenance like GLP-2. Their receptors *Glp2r* and *Igf1r*, but not *Igf2r*, seemed to increase in expression with TPN, perhaps as a compensatory mechanism, and fructose as well as allulose had no effect on the TPN-induced change (Figure 7). The gene expression levels of insulin-like growth factor binding proteins (Igfbps), which help in IGFs actions in peripheral tissues, were significantly decreased in the TPN group. Their levels were significantly but modestly recovered by fructose or allulose.

### 3.6. Effect of Allulose on Hormones Regulating Gut Differentiation and Barrier

**Figure 8:**
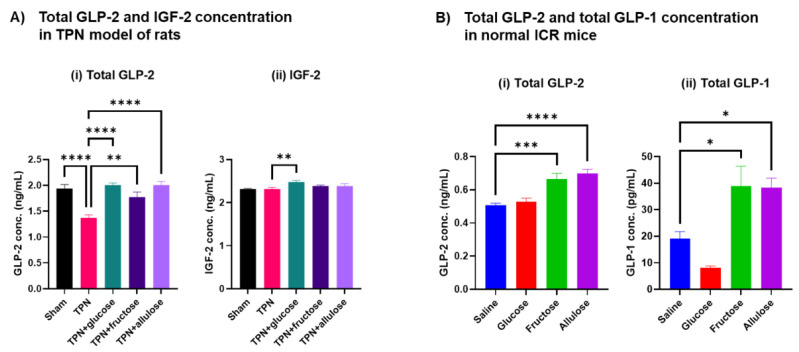
Concentration of GLP-2, IGF-2, and GLP-1 in plasma of peripheral blood in TPN model of rats and portal vein blood in normal mice. Measurement of total GLP-2 by EIA kit, IGF-2 by ELISA kit and total GLP-1 by ELISA kit in plasma. (**A**) Total GLP-2 (**i**) and IGF-2 (**ii**) concentration in plasma of peripheral blood in TPN model of rats. Values indicate mean ± SEM (*n* = 4). Asterisks indicate significant differences tested using the ANOVA-Dunnett’s multiple comparisons test (vs. TPN group, **** *p* < 0.0001, ** *p* < 0.01). (**B**) Total GLP-2 (**i**) and total GLP-1 (**ii**) concentration in plasma of portal vein blood in normal mice. Values indicate mean ± SEM (*n* = 6). Asterisks indicate significant differences tested using the ANOVA-Dunnett’s multiple comparisons test (vs. saline group, **** *p* < 0.0001, *** *p* < 0.001, * *p* < 0.05).

Because we found that oral administration of monosaccharide solution after TPN significantly altered the expression of hormonal pathway-related molecules associated with intestinal barrier function and epithelial cell maturation as well as the digestion and absorption of nutrients, we examined the GLP-2 secretion and the secretion of several hormones regulated by GLP-2 to investigate the underlying signaling pathways affecting functional changes throughout the jejunum. We measured GLP-2 concentrations which were involved in villous morphological and functional maintenance and GLP-2-associated hormones, IGF-2 in plasma by EIA for total GLP-2 or ELISA for IGF-2. First, we assessed whether monosaccharide supplementation can rescue the effect of TPN on total GLP-2 and IGF-2 concentration. IGF-2 concentrations were measured in this study because our previous studies have suggested that IGF-2 is involved in the maintenance of small intestinal villi function [22]. GLP-2 concentration decreased with TPN but was clearly rescued by monosaccharide administration. IGF-2 concentration was independent of treatment, suggesting that physiological responses to TPN may be driven primarily by GLP-2 (Figure 8A). We next examined whether these monosaccharides can directly induce GLP-2 secretion into the portal vein in normal physiological conditions. To verify this fact, we measured both GLP-2 and GLP-1 concentrations in the plasma of portal vein blood using normal mice. It has been reported that allulose promotes the secretion of GLP-1 [5,7]. We investigated to confirm whether a similar response to this report could be observed, and at the same time, whether fructose also promotes secretion of GLP-1. The concentration of gastrointestinal hormones in portal blood was measured 30 min after oral administration to mice of various monosaccharide solutions prepared to the same osmolality (30 mmol/kgBW, P.O.). GLP-2 and GLP-1 were significantly induced by oral administration of both fructose and allulose, but glucose administration was not induced by these hormones (Figure 8B). In summary, allulose and fructose seem to be potent inducers of GLP-1 and GLP-2, and glucose induces GLP-2 only when its concentration is reduced in TPN.

## 4. Discussion

Fructose, which has similar chemical structure to allulose, is metabolized in the body [27]. The specific action produced by fructose is considered to be due to fructose or its metabolites. In contrast, since it is not metabolized in the body, the physiological effects brought about by allulose are likely to be on allulose itself [12,28]. Fructose increases the expression of fructose-inducible genes GLUT5, G6Pase, SI and the like [29,30,31]. Additionally, dietary fructose and its metabolites change chromatin structure on upstream region of the GLUT5 gene [25], suggesting that chromatin remodeling is involved in the regulation of fructose-inducible genes. However, the mechanisms underlying the effect of allulose are unclear.

In this study, at least, it is revealed that luminal stimulation of allulose induces many gene expression levels involved in substance transport and nutrient metabolism that are expressed in intestinal epithelial absorptive cells. Furthermore, genes related to intestinal barrier functions such as Claudin 7 and tight junction proteins were markedly induced by allulose perfusion. Interestingly, the expression levels of these genes such as solute carrier family genes and tight junction related genes were hardly induced by fructose perfusion with the exception of the fructose-inducible gene such as Glut5. Additionally, although we investigated an influence of osmotic pressure against this phenomenon, osmotic stimuli from the luminal side did not affect the mRNA expression levels of representative genes when osmotic pressure was adjusted by the addition of 30 mM NaCl into KRB (data not shown). These results suggest that luminal allulose stimuli enhances not only the ability to digest and absorb nutrients, but also the intestinal barrier functions, and that the expression levels of these genes might be induced by luminal allulose-specific mechanism. On the other hand, among the categories of functions predicted to be downregulated by the IPA analysis were inflammatory responses, stress responses, and cell death. These functions are expected to have a negative effect for small intestinal tissues in the enhanced state, but are rather attenuated by allulose reflux. In fact, allulose has been reported to have antioxidant capacity in diabetic animals [15], and the anti-stress and anti-inflammatory effects of allulose cannot be ignored. Therefore, future studies should also analyze the mechanism of small intestinal function homeostasis focusing on the anti-stress and anti-inflammatory effects of allulose in the gastrointestinal tract.

We have previously shown that oral administration of fructose is more effective than oral administration of glucose in restoring small intestinal function [19]. The effect of dietary fructose intake on villi length elongation has been reported by Taylor et al. who showed that fructose and its metabolites act on pyruvate kinase isozymes M2 inactivation to enhance the viability of hypoxic intestinal epithelial cells [32]. In oncology, fructose supports tumor survival such as colon cancer, but in normal villi, it has been suggested that its action on villi elongation may contribute to increased nutrient absorption efficiency due to increased luminal surface area. Therefore, it is likely that a mechanism different from that of fructose regulates the expression of molecules that are specifically enhanced by allulose administration because allulose is not metabolized. Especially, intestinal barrier-related genes such as Tjp1, Ocln, Cldn7 and Cldn15, which is induced by allulose treatment, are molecules that maintain tighter tight junction and contribute to enhancing intestinal barrier function and suppressing inflammatory responses. Recently, the role of Cldn7 was mechanistically demonstrated by Cldn7 knockout mice; loss of Cldn7 promotes colitis and subsequent malignant transformation by disrupting tight junction integrity and increasing the inflammation [33]. Thus, it is suggested that allulose has an effect not only on the digestive and absorptive capacity of nutrients, but also on the suppression of inflammatory bowel disease (IBD). However, in this experiment, the small intestinal function-activating effects of allulose were lower than expected. There are two possible reasons for the negative results: First, because the small intestinal perfusion test involves continuous perfusion of a 60 mM monosaccharide solution for 5 h, the epithelial cells of the jejunal villi are constantly exposed to a constant dose of monosaccharide stimulation.

In the TPN experiment, on the other hand, monosaccharide stimulation occurs only once a day and the dose is 3 g/kg BW, which is not a large amount. Therefore, it suggested that the results of the TPN study were different from those of the small intestinal perfusion study. The second is that the small intestinal function-activating effects is considered to be affected by the time of administration of the monosaccharide solution. The turnover of small intestinal epithelial cells is estimated to be about 3 days. In other words, the villi are completely covered by the epithelial cells exposed to the stimuli only after being exposed to some stimuli for more than 3 days. In the present study, we used a 2-day administration condition to examine short-term effects, but in our previous study, we observed a marked recovery of small intestinal function after 3 days of administration. Therefore, it is suggested that even small doses can promote significant activation of small intestinal function by prolonging the administration period.

GLP-2 is a gastrointestinal hormone secreted from epithelial endocrine L cells, and its secretion is promoted in response to nutrient stimulation from the lumen [34,35,36]. GLP-2 then binds to GLP-2R in adjacent fibroblasts via paracrine signaling [37]. Fibroblasts stimulated by GLP-2 promote the secretion of various growth factors such as IGF, and act on respective receptors possessed on the surface of intestinal epithelial cells [38]. These hormonal actions are important for the maintenance of basic villous functions in the small intestine, and it is known to enhance the expression of molecules associated with nutrient digestion and absorption and intestinal barrier functions [39,40]. In addition, many of the basic functions of the small intestinal epithelial cells are regulated by feedback of GLP-2 stimulation via adjacent fibroblasts. Allulose has a secretagogue action of GLP-1 produced and secreted by the processing from the precursor of glucagon gene [41]. Through the same mechanism, enhancement of the secretion of GLP-2 may well be considered as the possibility that the basic function of the small intestine was transiently enhanced. In actually, we confirmed that GLP-2 secretion was induced by oral administration of both fructose and allulose in two animal studies, i.e., TPN study of rats and single ingestion study of mice. Until now, there have been no reports of GLP-2 secretion enhancement by allulose, and this is the first such report. However, there was no difference in the amount of GLP-2 secreted by each administered monosaccharide. These results suggest that secretion of GLP-2 is stimulated via a similar mechanism in response to stimulation of monosaccharide solution from the lumen.

On the other hand, it is known that IGF-2 is especially associated with cryptic cell proliferation via retinoblastoma (Rb) protein using Rb-deficient mice [42]. In addition, we reported that the villous atrophy in senescence-accelerated mouse SAMP10 strain may be involved in the IGF-2 signaling disorder [22]. In the fructose administration group following TPN treatment, where chorionic villi elongation was observed, we expected increased secretion of IGF-2, which is involved in the maintenance of villous morphology; however, IGF-2 was constantly secreted in peripheral blood of TPN treated rats. Most of the IGF-2 in peripheral blood is derived from the liver. Therefore, since the results of this study do not reflect IGF-2 secretion in the submucosa of the small intestine, it is necessary to measure IGF-2 secretion in portal blood. Several studies have reported that there is a link between GLP-2 secretion and IGF responsiveness, with effects that promote differentiation and maturation of small intestinal epithelial cells and cell proliferation in the crypts [43]. In particular, the presence of IGF-1 receptors has been recently reported to be important for the elongation of small intestinal villi [44,45]. In addition, the reactivity of its IGFs is significantly altered by the presence of IGFBPs, and IGFBP3 and IGFBP4 have been shown to play a key role in maintaining villi morphology and intestinal function [46,47]. These results suggest that the induction of expression of these genes enhanced the responsiveness of IGFs via stimulation of GLP-2 induced by fructose administration and resulted in maturation of small intestinal epithelial cells, such as marked villi elongation and the ability to digest and absorb nutrients. Interestingly, this reactivity is not seen with allulose administration and is specific to fructose administration. A characteristic response to allulose administration was an increase in lipids transport-related genes. It suggests that the increased expression of FABPs and lipoprotein APOA1 observed with allulose administration may be an enhancement via the GLP-2 pathway, because one study reported that GLP-2 increases intestinal lipid absorption and chylomicron production via CD36 [48]. Allulose treatment also specifically induced the expression of junctional adhesion molecule genes such as Tjp1, Ocln, and Cldn15 genes; the significant increase in the expression of these genes, which was not seen in the fructose-treated group, suggested the involvement of other intracellular signals other than GLP-2 signaling pathway, such as Cdx-2, HNF-1, and HNF-4, and SP1, suggesting the involvement of other multiple transcriptional factors. In particular, HNF-4 was extracted by IPA analysis as a candidate upstream regulator affected by allulose stimulation. However, the results of this experiment lack evidence to explain the above ideas. Therefore, the characterization of the nutritional effects of fructose and allulose administration on the homeostasis of small intestinal villi function, respectively, not only of common mechanisms of action, such as the association of IGFs and IGFRs via GLP-2 stimulation, but also of other regulatory mechanisms that characterize each action. The presence of other regulatory mechanisms that characterize the actions of each should be analyzed in more detail at the protein levels.

## 5. Conclusions

The mechanism of the surprising effect of allulose on the gene expression in small intestinal epithelial cells is still unclear. However, this study is the first to demonstrate that allulose, which is not metabolized by the body, has the ability to maintain not only expression of genes involved in a wide variety of functions but also structure of crypts and villi, which is similar to or exceeds that of widely consumed sugars, glucose and fructose, considered risk factors of metabolic disease. Thus, we need to demonstrate in further investigation that continuous allulose intake can be a useful tool to maintain or recover the gastrointestinal capabilities. In the future, we plan to investigate the anti-inflammatory effects of allulose and whether it has beneficial effects on some intestinal diseases, such as inflammatory bowel disease and leaky gut, which we did not focus on in this study.

## Figures and Tables

**Table 1:** (**A**) Pathways and gene clusters predicted to be upregulated by allulose perfusion compared to glucose and to fructose. (**B**) Pathways and gene clusters predicted to be downregulated by allulose perfusion compared to glucose and to fructose.

**A**					
**Categories**	**Diseases or Functions Annotation**	**Activation z-Score**			**#Molecules**
		**F/G**	**A/G**	**A/F**	
Cancer, Organismal Injury and Abnormalities	Advanced extracranial solid tumor	2.13			100
	Advanced malignant tumor			2.13	174
	Secondary tumor			2.13	163
	Advanced lung cancer	2.13			51
Carbohydrate Metabolism	Uptake of carbohydrate		2.92	2.42	56
	Uptake of monosaccharide		2.43		52
Carbohydrate Metabolism, Lipid Metabolism, Small Molecule Biochemistry	Metabolism of phosphatidic acid			2.41	41
	Synthesis of phosphatidic acid			2.00	37
Carbohydrate Metabolism, Molecular Transport, Small Molecule Biochemistry	Uptake of D-glucose		2.47	2.47	47
	Uptake of D-hexose		2.59	2.32	48
Cell Death and Survival, Organismal Injury and Abnormalities	Cell death of cervical cancer cell lines		2.40	2.21	71
	Cell death of epithelial cell lines		2.15		55
Cell Morphology, Cellular Assembly and Organization, Cellular Function and Maintenance	Formation of cellular protrusions		2.55	2.82	142
Cell-To-Cell Signaling and Interaction	Adhesion of tumor cell lines		2.29	2.63	55
	Binding of tumor cell lines		3.08	3.39	72
	Interaction of tumor cell lines		2.70	3.26	76
Cellular Assembly and Organization, Cellular Function, Maintenance and Tissue Development	Development of cytoplasm		2.48	2.70	96
	Formation of cytoskeleton		2.25	2.49	67
	Formation of membrane ruffles		2.40		17
	Microtubule dynamics			2.10	182
	Formation of actin filaments		2.28	2.53	57
	Formation of actin stress fibers			2.01	47
	Fibrogenesis		2.42	2.20	79
	Formation of filaments		2.35	2.41	72
Cellular Compromise, Inflammatory Response	Degranulation of cells		2.03	2.42	108
	Degranulation of phagocytes		2.27	2.27	96
Cellular Function and Maintenance	Endocytosis		3.36	3.36	94
	Engulfment of cells		3.41	3.41	83
	Internalization by tumor cell lines		2.41	2.74	27
Cellular Function and Maintenance, Inflammatory Response	Phagocytosis		3.63	3.63	58
Infectious Diseases	HIV infection			2.07	120
	Infection by HIV-1			2.03	104
	Infection by Retroviridae		2.22	2.37	124
	Infection by RNA virus		2.43	2.57	155
	Infection of cells		2.26	2.40	140
	Infection of cervical cancer cell lines		2.50	2.69	78
	Infection of tumor cell lines	2.13	2.84	3.02	92
	Viral Infection		2.56	2.63	294
Lipid Metabolism, Small Molecule Biochemistry	Fatty acid metabolism			2.05	112
	Metabolism of membrane lipid derivative		2.64	3.17	99
Molecular Transport	Secretion of molecule		2.94	2.74	103
	Transport of molecule		4.48	4.17	305
Organismal Development	Size of body		2.90	2.90	131
Post-Translational Modification	Ubiquitination			2.34	70
	Ubiquitination of protein	2.01		2.01	69
Protein Trafficking	Interaction of protein		2.24	2.24	48
**B**					
**Categories**	**Diseases or Functions Annotation**	**Activation z-Score**			**Molecules**
		**F/G**	**A/G**	**A/F**	
Cardiovascular Disease, Hematological Disease, Organismal Injury and Abnormalities	Anemia		−2.49	−2.49	74
Cell Cycle, Cellular Movement	Cytokinesis of tumor cell lines	−2.05	−2.05		17
Cell Death and Survival	Apoptosis of breast cancer cell lines	−2.03			61
Cellular Compromise	Stress response of cells	−2.02			28
	Stress response of tumor cell lines	−2.58	−2.05	−2.05	15
Cellular Development, Cellular Growth and Proliferation, Connective Tissue Development and Function, Tissue Development	Cell proliferation of fibroblasts	−3.09			72
Connective Tissue Disorders, Inflammatory Disease, Inflammatory Response, Organismal Injury and Abnormalities, Skeletal and Muscular Disorders	Inflammation of joint		−2.39	−2.39	153
Connective Tissue Disorders, Inflammatory Disease, Organismal Injury and Abnormalities, Skeletal and Muscular Disorders	Rheumatic Disease		−2.21	−2.21	199
Free Radical Scavenging	Generation of reactive oxygen species	−2.05			42
Inflammatory Response	Inflammation of absolute anatomical region		−2.23	−2.23	181
	Inflammation of body cavity		−3.05	−3.05	152
Inflammatory Response, Organismal Injury and Abnormalities	Inflammation of organ		−2.43	−2.43	219
Lipid Metabolism, Molecular Transport, Small Molecule Biochemistry	Concentration of fatty acid		−2.5	−2.12	52
Neurological Disease	Motor dysfunction or movement disorder		−2.13	−2.53	185
	Movement Disorders		−2.04	−2.45	182
Organismal Injury and Abnormalities, Renal and Urological Disease	Urination disorder		−3.24	−3.55	54
Organismal Survival	Morbidity or mortality		−3.12	−3.12	402
	Organismal death		−3.19	−3.19	396
Protein Synthesis	Expression of protein		−2.57	−2.57	80

## Supplementary Materials

The following supporting information can be downloaded at: www.mdpi.com/article/10.3390/nu14153230/s1, Table S1: Primers sequences for qRT-PCR.
