# Supplementary material for: Comparative Effects of Allulose, Fructose, and Glucose on the Small Intestine
Source: Nutrients. 2022 Aug 7;14(15):3230. doi: 10.3390/nu14153230 (PMC9370476; doi:10.3390/nu14153230)
Supplement: Supplementary file 1 [file nutrients-14-03230-s001.zip › nutrients-1851443-supplementary.pdf]

Table S1. Primers sequences for qRT-PCR.

Accession No.	Gene Name	Primer sequence	Product size (bp)
NM_001004198.1	TATA box binding protein (*Tbp*)	5'-ATAATCCCAAGCGGTTTGCTG-3'<br>5'-TGCACACCATTTTCCCAGAAC-3'	90
NM_013061	Sucrase-isomaltase（*Si*）	5'-GCAGAAGGCTACATGGA-3'<br>5'-CCTTGCGACTGTCTCA-3'	141
NM_053841.1	Lactase (*Lct*)	5'-GATGAGCTATGCAGGCTATGG-3'<br>5'-ATGTGCCTTGAGGATCAGGT-3'	100
NM_023963.2	Caudal type homeobox 2 (*Cdx2*)	5'-TGGTGTACACAGACCATCAGC-3'<br>5'-CTGCGGTTCTGAAACCAAAT-3'	142
NM_13033.2	Sodium-glucose co-transporter (*Sglt1*)	5'TGTACCCTGTGTGGCT—3'<br>5'-GGTGCCGCAGTATTTC-3'	132
NM_012879.2	Glucose transporter 2 (*Glut2*)	5'-CTTGGTTCATGGTTGCTGAA-3'<br>5'-AAATTGCAGACCCAGTTGCT-3'	98
NM_031741.1	Facilitated fructose transporter (*Glut5*)	5'-GTCTTGGCAAGCGAGGAA-3'<br>5'-CAGGAAAGAAGGGCAGCA-3'	100
NM_12556.2	Fatty acid binding protein 1 , liver (*Fabp1*)	5'-CCAAGTGCAGAGCCAAGAGA-3'<br>5'-CCCATAGGTGATGGTGAGTTTG-3'	143
NM_013068.1	Fatty acid binding protein 2 , intestinal (*Fabp2*)	5'-GGCATTAACGTGGTGAAGAGG-3'<br>5'-GACGCCGAGTTCAAACACAA-3'	135
NM_001106266	Tight junction protein 1 (*Tjp1*)	5'-GCCGAAGCCAGTCACGATCT-3'<br>5'-TCTGCTCCGGGAGACTGCCAT-3'	98
NM_053773.2	Tight junction protein 2 (*Tjp2*)	5'-CGCTGAAGACCGCATGTCCT-3'<br>5'-TCCCTCGCCGTCCGTATCTT-3'	100
NM_001108073.1	Tight junction protein 3 (*Tjp3*)	5'-GCGGAGGACCAGCTGAATGG-3'<br>5'-CGCTGTTAGCCCGACTGTCA-3'	103
NM_001012022	Claudin 3 (*Cldn3*)	5'-CACACCGCACCATCACCACT-3'<br>5'-ACTGCGTGGCGTCTGTAACC-3'	109
NM_031702	Claudin 4 (*Cldn4*)	5'-AGCCGTGTTCATCGTGGCAA-3'<br>5'-CGGAAGCCACCAGAGGGTTG-3'	98
NM_001107135	Claudin 7 (*Cldn7*)	5'-CGTGGCAGGTCTTGCTGCTT-3'<br>5'-AGACCCTGCCCAGCCGATAA-3'	135
NM_031700	Claudin 15 (*Cldn15*)	5'-TGGGCGGCATCTGTGTCTTC-3'<br>5'-GAAGTGGCTCGGGCTGTCAC-3'	112
NM_031329	Occludin (*Ocln*)	5'-CGCACGAGGTCTGCTTGTCT-3'<br>5'-CCGGCCTGTAAGGAGGTGGA-3'	118
NM_012736	Apolipoprotein A-1 (*Apoa1*)	5'-TCAGGAGTTCTGGGCTAACCTG-3'<br>5'-ACCTCCTCGTTCCACTTCTCCT-3'	132
NM_012737	Apolipoprotein A-4 (*Apoa4*)	5'-TGTTCCTGAAGGCTGTGGT-3'	94

		5'-CACATCACATTGGCCACCT-3'	
NM_019287	Apolipoprotein B (*Apob*)	5'-AGCGCCACCAAGATTAACTG-3'<br>5'-CATGATGAGAGTGCAGACTTGG-3'	63
NM_012501	Apolipoprotein C3 (*Apoc3*)	5'-CTAAGCAGCATGCAGGAGTCT-3'<br>5'-CAGGGATTTGAAGCGATTG-3'	72
NM_021848.1	Glucagon-like peptide 2 receptor (Glp2r)	5'-CGGTATGTGTGCTGGCCTCA-3'<br>5'-GCAGTGTCTGTAGGCCCTTCC-3'	107
NM_052807.2	Insulin-like growth factor 1 receptor (Igf1r)	5'-CGAAAACCATCGATTCTGTG-3'<br>5'-CCAATTCCGAGGCAATGTTA-3'	111
NM_012756.2	Insulin-like growth factor 2 receptor (Igf2r)	5'-TGGAAAGCTGTCCTTAGATGTGT-3'<br>5'-CGAAAGTCAGCTTGATTAAGG-3'	128
NM_013144.1	Insulin-like growth factor binding protein1 (Igfbp1)	5'-TGCCATTAGCACCTACAGCA-3'<br>5'-AACACTTTATAGAGTTCCCGTTGG-3'	93
NM_012588.2	Insulin-like growth factor binding protein3 (Igfbp3)	5'-GCCAGCGCTACAAAGTTGAC-3'<br>5'-GTGTGTCCTCCATTTCTCTGC-3'	114
NM_001004274.2	Insulin-like growth factor binding protein4 (Igfbp4)	5'-CAACTTCCACCCCAAACAGT-3'<br>5'-GATCCACACACCAGCATTTG-3'	68
